# Targeting G6PD (Glucose-6-Phosphate Dehydrogenase) as a Biomarker of Therapeutic Vulnerability in Renal Cell Carcinoma

**DOI:** 10.3390/ijms27062844

**Published:** 2026-03-20

**Authors:** Daniel Pinheiro Ferreira, Ana Carolina Souza Mizael, Julia Victória Bonifácio Cabrieira, Rafaela Viviane Neves Silva, Liliane Silvano Araújo, Crislaine Aparecida Silva, Karen Bento Ribeiro, Adilha Misson Rua Michelleti, Juliana Reis Machado, Régia Caroline Peixoto Lira

**Affiliations:** 1 Clinics Hospital, Universidade Federal do Triângulo Mineiro, Uberaba 38025-180, Brazil; daniel.uro@hotmail.com (D.P.F.); kkarenbento@yahoo.com.br (K.B.R.); 2 Department of Pathology, Genetics and Evolution, Universidade Federal do Triângulo Mineiro, Uberaba 38025-015, Brazil; anacarol.acm36@gmail.com (A.C.S.M.); julia.vbonifacioc@gmail.com (J.V.B.C.); juliana.machado@uftm.edu.br (J.R.M.); 3 Kidney Research Center, Universidade Federal do Triângulo Mineiro, Uberaba 38025-015, Brazil; rafaelaviviane912@gmail.com (R.V.N.S.); liliane.araujo@uftm.edu.br (L.S.A.); crislaine.silva@uftm.edu.br (C.A.S.); 4 Department of Clinical Surgery, Universidade Federal do Triângulo Mineiro, Uberaba 38025-440, Brazil; adilha.micheletti@uftm.edu.br

**Keywords:** G6PD, renal cell carcinoma, tumor aggressiveness, therapeutic target

## Abstract

Renal cell carcinoma (RCC) is the most common kidney cancer, with increasing global incidence. Despite advances with VEGF-targeted tyrosine kinase inhibitors (TKIs) and immunotherapies, therapeutic resistance remains frequent, limiting long-term benefits. This highlights the need for potential biomarkers of tumor aggressiveness and therapeutic candidates, such as glucose-6-phosphate dehydrogenase (G6PD), whose altered expression has been associated with several cancers. We evaluated *G6PD* gene and protein expression in 121 RCC samples through immunohistochemistry and assessed functional role *in vitro* approaches. 786-O and ACHN cells were treated with the inhibitor G6PDi-1 and the anti-VEGF cabozantinib/lenvatinib. *G6PD* mRNA levels were higher in tumors than in non-neoplastic tissues, indicating shorter overall survival in clear cell (ccRCC) and papillary (pRCC) subtypes. Immunolabeling confirmed a higher expression in pRCC and associations with pathological features. CRISPR and RNAi datasets revealed a stronger G6PD dependency in the ccRCC. A high gene expression was observed in lenvatinib non-responder cell lines, and DepMap dose–response curves indicated modest responses to VEGF inhibitors. *In vitro*, ACHN was more sensitive to VEGF inhibition, particularly cabozantinib, whereas G6PDi-1 had stronger effects in 786-O, impairing viability, migration, and clonogenic capacity. Our findings support G6PD as a biomarker of tumor aggressiveness and G6PDi-1 as a potential therapeutic in RCC models.

## 1. Introduction

Renal cell carcinoma (RCC) is the most common type of kidney cancer, accounting for 2–3% of all cancer diagnoses [1]. The clear cell histological subtype (ccRCC) is the most common, comprising 75–80% of cases, followed by papillary (pRCC) and chromophobe RCC (chRCC). Prognosis is based on clinical and pathological features, including TNM staging, tumor size, Fuhrman grade, necrosis, and histological subtype. Despite these criteria, about 30% of patients present with metastasis at diagnosis, and another 30% experience recurrence after treatment. Currently, no molecular biomarker is routinely used to predict tumor aggressiveness or outcome [2,3,4,5].

Despite therapeutic advances, including PD-L1 inhibitor immunotherapies, the vascular endothelial growth factor (VEGF) remains a key molecular target in RCC treatment [6]. Under physiological conditions, VEGF stimulates the formation of new blood vessels, ensuring adequate oxygenation and tissue nutrition. In the tumor context, the pathway promotes pathological angiogenesis, contributing to tumor invasion, metastasis, and disease progression [7]. Inhibitors such as cabozantinib and lenvatinib are widely used, often in combination with immunotherapies [8]. Nonetheless, the tumor resistance to standard protocols remains an important clinical challenge, limiting the long-term efficacy of these strategies and the gains in survival.

In light of the need for new therapeutic targets, glucose-6-phosphate dehydrogenase (G6PD) has been identified as a potential biomarker, being overexpressed in distinct types of cancer, such as ovarian [9], breast [10], cervical [11], prostate [12], bladder tumors [13] and RCC [14,15]. In general, tumor cells reprogram their energy metabolism to sustain high rates of proliferation, and one of the commonly altered pathways is the pentose phosphate pathway, whose first step is catalyzed by the G6PD enzyme. The main objective is to produce essential nucleotide precursors for the replication, transcription, and translation of genetic material, sustaining cell proliferation and protein expression. In addition, nitrogenous compounds, important for cellular metabolism, are produced after G6PD activity, especially the NADP+/NADPH cofactor, which maintains cellular antioxidant systems mediated by the glutathione enzyme [16]. Under normal conditions, high levels of ATP and glucose-6-phosphate can act as positive modulators of G6PD activity, triggering signals for cell proliferation or nitrogen compound synthesis [17]. In malignant cells, this metabolic regulation can be impaired, leading to G6PD overexpression and constitutive activity. As a result, cancer cells become more resistant to oxidative damage, while simultaneously enhancing the production of nucleotides and enzymatic cofactors that are essential for tumor growth and progression [18].

In this study, we aimed to evaluate G6PD as a biomarker of aggressiveness and potential therapeutic vulnerability in renal cell carcinoma. Specifically, G6PD expression was investigated across the three main tumor subtypes and was associated with pathological features. We further explored the functional impact of G6PD expression and inhibition, comparing their effects with those of VEGF-targeted therapies in RCC cell line models.

## 2. Results

### 2.1. G6PD High Expression Suggests RCC Aggressiveness and Therapy Response

**Figure 1:**
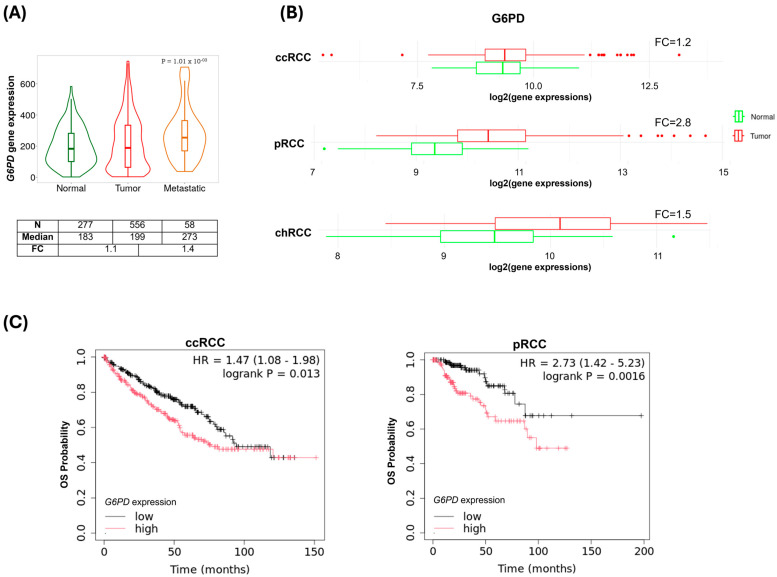
*G6PD* gene expression pattern in kidney samples. (**A**) *G6PD* levels in normal, tumor, and metastatic samples (Gene-chip database, Kruskal–Wallis test, TNMplot tool). (**B**) Expression profile in normal kidney (green) and tumors (red) according to the histological subtypes (ccRCC) clear cell, (pRCC) papillary and (chRCC) chromophobe (RNA-seq dataset, Multi-gene analysis tool). (**C**) Overall survival (OS) according to gene expression in ccRCC and pRCC (RNA-seq database, KM-plotter tool, cut-off: median). N: number of samples; FC: fold change; HR: hazard ratio.

To assess the relevance of G6PD in tumor prognosis and aggressiveness, we conducted gene expression (*in silico*) and protein expression (immunohistochemistry) analyses in human tumor and non-tumor samples. Elevated *G6PD* levels were found in metastatic RCC specimens compared to primary tumors and normal samples (FC = 1.4); see Figure 1A. All histological subtypes presented higher expression than normal kidney tissues, being more prominent for pRCC (FC = 2.8) and chRCC (FC = 1.5); see Figure 1B. Moreover, *G6PD* upregulation was significantly associated with shorter overall survival for ccRCC and pRCC; see Figure 1C. Corroborating the gene expression observations, all normal renal tissues were negative for G6PD in immunohistochemistry. The RCC cohort consisted of 69.5% of T1-T2 stages, 59.5% of Fuhrman 1–2 (59.5%), 55.7% presenting tumor necrosis, only 4.6% of positive lymph nodes, 9.2% presenting metastasis, 5.3% of tumor recurrence (average of 32.71 months), and 13.7% of death (Supplementary Materials S1). Most of the tumor samples (80.2%) were negative for G6PD; 11.6% presented weak intensity, 2.5% were moderate, and only 5.8% showed strong immunolabeling with cytoplasmic staining (Supplementary Figure S1).

**Figure 2:**
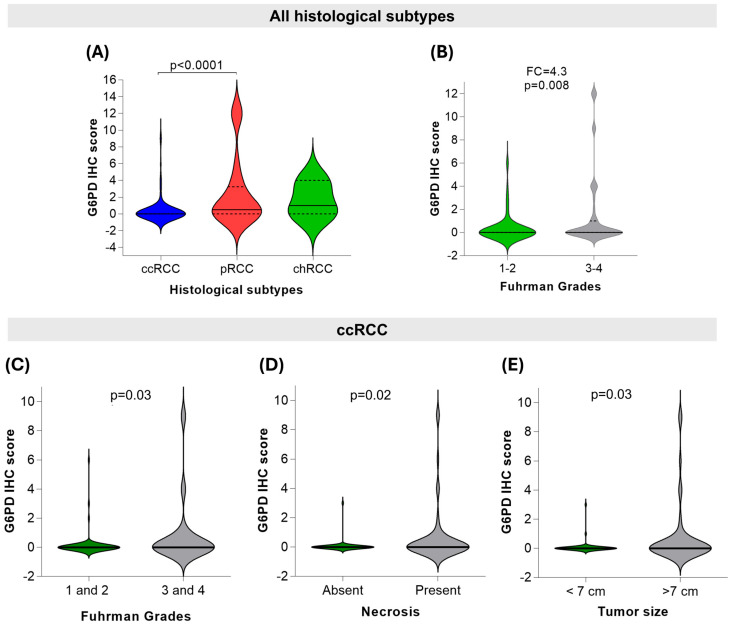
G6PD protein expression according to RCC pathological features. (**A**) Immunohistochemistry score of G6PD in the distinct histological subtypes. (**B**) Expression score according to Fuhrman grades. (**C**–**E**) G6PD score in the clear cell RCC subtype according to Fuhrman grade, tumor size, and the presence of necrosis, respectively.

G6PD-positive cells were predominant in women, in pRCC and chRCC histological subtypes, and in tumors with higher Fuhrman grades and above seven centimeters (Supplementary Table S1). The immunostaining score was significantly higher in pRCC compared to ccRCC (Figure 2A), and in grades 3–4 compared to 1–2 (Figure 2B). When analyzing only the ccRCC subtype, the G6PD protein expression sustained elevated scores associated with Fuhrman 3–4 (Figure 2C) and tumors larger than 7 cm (Figure 2D), in addition to the presence of necrosis (Figure 2E).

**Figure 3:**
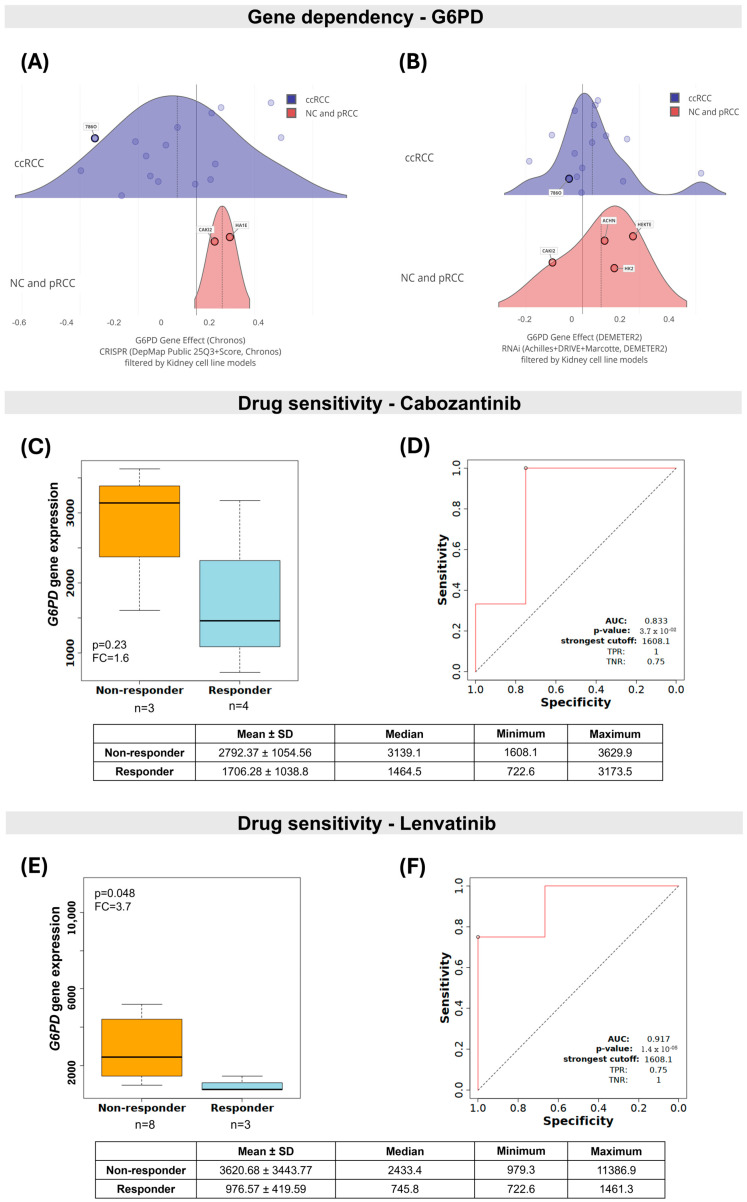
*G6PD* dependency and response to targeted therapies in renal cell carcinoma cell lines. Effect of *G6PD* gene inhibition in clear cell renal cell carcinoma (ccRCC, purple) compared with non-cancerous (NC) and papillary renal cell carcinoma (pRCC, red) cell lines, based on Cancer Dependency Map (DepMap). (**A**) CRISPR assays (Chronos) and (**B**) RNAi screens (DEMETER2). A lower score means that the gene is more likely to be dependent on a given cell line. The more negative the score, the greater the cell line’s dependence on the gene. A score of 0 is equivalent to a gene that is not essential, whereas a score of −1 corresponds to the median of all common essential genes. (**C**–**F**) *G6PD* gene expression in cabozantinib and lenvatinib responder and non-responder renal cancer cell lines stratified by IC50 tertiles (DepMap dataset), and their respective Receiver Operating Characteristic (ROC) curve analyses. AUC: Area Under the Curve; FC: fold change; TPR: True Positive Rate; TNR: True Negative Rate.

A gene dependency analysis based on CRISPR and RNAi datasets was performed across cell lines to evaluate dependency on *G6PD*. The results revealed that ccRCC cell lines, notably 786-O, are more dependent on *G6PD* expression than non-cancerous (HA1E, HEKTE, HK2) or pRCC (ACHN, CAKI2) cells (Figure 3A,B). Additionally, elevated levels of *G6PD* were observed in non-responders kidney cancer cells treated with cabozantinib (FC = 1.6, *p* = 0.23) and lenvatinib (FC = 3.7, *p* = 0.048); see Figure 3C,E. ROC curve analysis further supported these associations, demonstrating a significant capacity of *G6PD* expression to discriminate non-responder from responder cells for both therapies (cabozantinib: AUC = 0.83, *p* = 0.037; lenvatinib: AUC = 0.92, *p* = 0.000014); see Figure 3D,F.

### 2.2. G6PD Inhibition Is More Effective than Standard Therapies in Specific RCC Subtype Cell Lines

**Figure 4:**
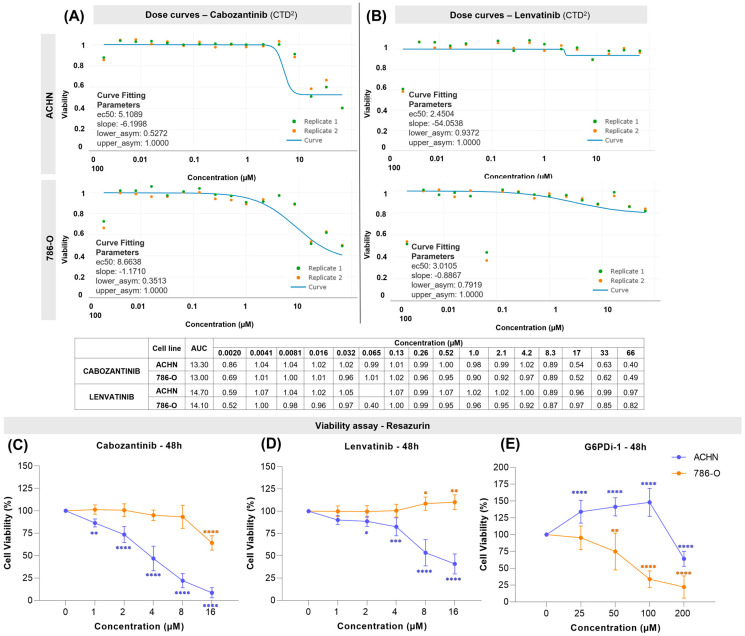
Cell sensitivity to cabozantinib, lenvatinib, and G6PD-1. (**A**,**B**) Dose–response curve from CTD2 DepMap dataset analyses for cabozantinib and lenvatinib using ACHN and 786-O cell lines. EC50: Effective Concentration 50%. (**C**–**E**) Comparison of cell viability between ACHN (blue line) and 786-O (orange line) through resazurin assay after 48 h of treatment with (**C**) cabozantinib, (**D**) lenvatinib, and (**E**) G6PDi. Statistical significance is indicated as follows: * *p* ≤ 0.05, ** *p* ≤ 0.01, *** *p* ≤ 0.001, and **** *p* ≤ 0.0001 compared to the control group (0 µM).

To compare the effects of G6PD inhibition with two drugs that are established clinical treatments, we performed complementary *in silico* and *in vitro* analyses using RCC cell line models. Drug sensitivity analysis based on the AUC (CTD^2^) dataset was used to provide an initial comparative assessment of the effects of cabozantinib and lenvatinib on RCC cell viability. The results indicated that treatment with cabozantinib (0.0020 µM to 66 µM) reduces the cell viability of ACHN (EC50: 5.1089; Slope: −6.1998) and 786-O (EC50: 8.6638; Slope: −1.1710) in a dose-dependent manner, while lenvatinib exerts a limited effect: ACHN (EC50: 2.4504; Slope: −54.0538) and 786-O (EC50: 3.0105; Slope: −0.8867). In Figure 4A (cabozantinib) and Figure 4B (Lenvatinib), although the numerical values appear similar, the dose–response curves indicate a greater reduction in cell viability in the ACHN cell line, compared to 786-O, as supported by the EC50 values. It is worth noting that the response curves have not reached a 50% reduction in cell viability at the concentrations evaluated. Secondary repurposing screening analyses from PRISM corroborate these findings for both drugs (Supplementary Figure S2).

These findings were subsequently validated using resazurin-based cell viability assays, along with clonogenic and migration assays using two well-characterized RCC cell lines (ACHN and 786-O). At this point in the study, we were able to understand the effects of pharmacological G6PD inhibition better and compare them with those observed with VEGF inhibitors. Consistently, the resazurin assay showed that cabozantinib has a more pronounced effect than lenvatinib after 48 h of treatment. Particularly in ACHN, cell viability was decreased by 91.3% at the highest dose (IC_50_: 5.40 µM; 95% CI: 5.22–5.59), whereas it only reached a 35.8% reduction in 786-O (IC_50_: 19.31 µM; 95% CI: not calculable); see Figure 4C. Lenvatinib did not affect 786-O, but impaired ACHN cell viability by 59% (IC_50_: 11.46 µM; 95% CI: 10.19–13.08); see Figure 4D. Contrarily, the G6PD inhibition using G6PDi-1 significantly decreased the cell viability of 786-O from 50 µM on, reaching a 78% reduction (IC_50_: 105.2 µM; 95% CI: 86.99–129.8). In ACHN cells, G6DPi produced a modest maximal effect of 36% (IC_50_ could not be estimated). A measurable reduction in viability was observed only at the highest concentration, whereas lower concentrations seem to increase resazurin metabolism; see Figure 4E. Treatment with G6PDi-1 and VEGF inhibitors for 24 h resulted in slight reductions in viability in both cell lines (Supplementary Figure S3).

**Figure 5:**
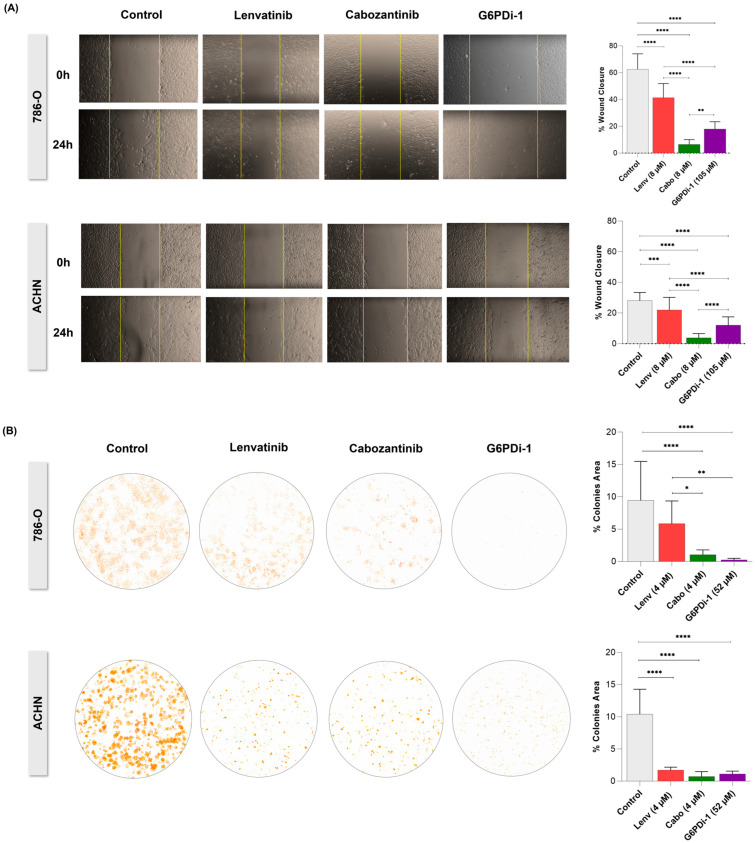
Effects of VEGF inhibitors (cabozantinib and lenvatinib) and G6PDi-1 on migration and clonogenic capacity of RCC cells. (**A**) Representative images of wound healing at 0 h and 24 h, followed by graphs showing the percentage of gap closure for each treatment (controls: 62.6% for 786-O and 28.1% for ACHN). (**B**) Representative images of the clonogenic assay, accompanied by the respective graphs quantifying the clonogenic area for each experimental condition (controls: 9.4% for 786-O and 10% for ACHN). Statistical significance is indicated as follows: * *p* ≤ 0.05, ** *p* ≤ 0.01, *** *p* ≤ 0.001, and **** *p* ≤ 0.0001 compared to the control group (0 µM).

Cabozantinib also produced the greatest impact on cell migration impairment (6.4% of wound closure in 786-O and only 3.7% in ACHN) compared to lenvatinib (41.3% of closure in 786-O and 22% in ACHN) and even to G6PDi-1, which exerted an intermediate effect (18% of gap closure in 786-O and 12% in ACHN); see Figure 5A. The antitumoral potential of G6PDi-1 in 786-O cells was reinforced by decreasing the clonogenic area to 0.2%, while cabozantinib and lenvatinib reduced it to 1% and 5.8%, respectively; see Figure 5B. In the ACHN, all inhibitors presented similar results, reducing the colony area to 1.1% (G6PDi-1), 0.7% (cabozantinib), and 1.7% (lenvatinib); see Figure 5B.

## 3. Discussion

Metabolic adaptation in cancer underscores the relevance of biomarkers such as G6PD, a key enzyme of cell proliferation and the redox homeostasis, that produces most of the NADPH in the cell [19]. Its upregulation has been reported in different human cancers compared to normal counterparts [9,10,11,12,13,20], and it was suggested as an important prognostic indicator for gastric cancer [21], breast carcinoma [10,22] and renal cell carcinoma [14,15].

Previous studies have revealed associations between *G6PD* gene overexpression and the advanced pathological stages of ccRCC and pRCC, as well as ccRCC unfavorable outcome and Fuhrman grades [3,4,15,23]. Accordingly, in our cohort, we observed higher *G6PD* levels in metastasis than in primary tumors and normal samples, being more prominent in pRCC and predicting shorter overall survival for both RCC subtypes. Given that G6PD is a promising prognostic biomarker, its protein expression in our local samples of RCC, with the majority of ccRCC, is in accordance with the RCC worldwide incidence; higher levels of protein were associated with larger tumors, advanced Fuhrman grades, and a potential role of G6PD in aggressive behavior [24]. All adjacent non-neoplastic tissues and most tumors with favorable prognosis profiles were negative for G6PD.

According to the American Joint Committee on Cancer (AJCC) guideline, the Fuhrman classification is a relevant prognostic factor based on nuclear morphological alterations, which was validated for ccRCC and pRCC [25]. The original publication has reported a 5-year survival rate of 64% for grade 1, 34% for grade 2, 31% for grade 3 and 10% for grade 4 [26]. Although G6PD has been only sparsely explored across RCC histological subtypes, a study including 74 tumors reported no clear differences in the protein expression among RCC subtypes [27], contrasting with our findings, as we observed a higher G6PD expression in the papillary subtype.

Specifically, in the ccRCC subtype, elevated G6PD protein expression in our samples remained associated with tumor size and Fuhrman grade, in addition to the presence of necrosis. Tumor necrosis has been described as an independent predictor of survival, particularly in patients with ccRCC, which reinforces the importance of our finding, given that it suggests G6PD as a biomarker of the tumor aggressiveness [28,29,30]. A study involving 149 ccRCC patients also reported significant associations between high G6PD protein levels and Fuhrman grades 3–4, tumor extent (T), lymph node metastasis, TNM stage, and poor clinical outcomes [15]. In our cohort, no differences in metastasis or overall survival were observed, likely due to the low number of metastatic cases and deaths. We also found a higher frequency of G6PD-positive cells in female samples, which contrasts with a previous report on ccRCC [15]. As the *G6PD* gene is located on the X chromosome (*Xq28*), sex-related biological differences such as X-inactivation and hormones may influence its expression and activity [31,32,33]. Importantly, G6PD expression may also be affected by tumor metabolic demands and tumor microenvironmental factors. This consideration becomes particularly relevant for further discussion, since men are more frequently affected by RCC and the male gender is usually associated with an aggressive tumor profile [34,35].

Experimental evidence suggests that G6PD promotes RCC progression by enhancing cell proliferation and invasion [27,36]. In melanoma cells, *G6PD* suppression reduces proliferation, increases apoptosis, and reverses chemoresistance [37,38]. Together, these previous findings support the hypothesis that a hyperactivation of the pentose phosphate pathway boosts nucleotide and NADPH production, which leads to heightened proliferative capacity in tumor cells and increased resistance to oxidative stress induced by chemotherapy [39,40,41]. Consistently, our *in silico* analyses revealed that ccRCC cell lines, particularly 768-O, appear to have a greater dependency on the *G6PD* gene than cells with a pRCC profile or non-cancerous cells. This dependency profile may explain the aggressive behavior of this cell model and ccRCC subtype, since the G6PD supports mechanisms that enhance the cellular ability to survive and adapt, even under unfavorable or stressful microenvironment. In addition, we observed higher levels of *G6PD* in cell lines resistant to lenvatinib and cabozantinib, which are two tyrosine kinase inhibitors (TKIs) approved as first- or second-line therapies for advanced RCC [24].

Cabozantinib was approved based on its ability to achieve longer median progression-free survival compared with everolimus (7.4 vs. 3.8 months) [42], while the approval of lenvatinib plus everolimus was supported by its superior progression-free survival (14.6 months) relative to everolimus alone (5.5 months) or lenvatinib alone (7.4 months) [43]. Although lenvatinib and cabozantinib share VEGFR-related targets, they are used in different dosing strategies and combination regimens in clinical practice. To address the impact of both drugs in experimental RCC models, we used cell lines with molecular profiles of the two most common histological subtypes: ccRCC (786-O) and pRCC (ACHN). Through *in silico* investigation, we observed a general resistant profile in both cell lines, indicating that increased concentrations or therapeutic combinations are necessary to achieve the desired cytotoxic effect. Our *in vitro* analyses confirmed a dose-dependent effect and demonstrated that cabozantinib has a greater antitumor effect than lenvatinib, with ACHN demonstrating the highest sensitivity. Similar results have been reported in hepatocellular and oral squamous cell carcinoma models, in which both drugs have reduced cell viability with the same dose-dependent pattern [44,45,46].

The greater sensitivity of ACHN to cabozantinib may reflect the drug’s mechanism of action and the cell line molecular profile. Although cabozantinib has a multi-kinase inhibitory profile, targeting additional pathways associated with tumor survival and therapeutic resistance, it potently inhibits MET and VEGFR2, which are widely involved in tumor pathogenesis [47]. Interesting, ACHN cells harbor activating MET mutations that promote tumor growth and cell survival signals, making them particularly vulnerable to MET blockade [48,49]. Together, this broader inhibition spectrum and the cell profile may contribute to the observed biological effects.

In contrast, lenvatinib does not directly target MET, which may explain its lower efficacy in ACHN cells [48,49]. In parallel, the reduced sensitivity of 786-O cells to cabozantinib may be associated with the *VHL* mutation, which stabilizes HIF and sustains VEGFR signaling. These metabolic features reflect a more aggressive tumor profile and may limit TKI efficacy. Clinically, ccRCC patients frequently develop TKI resistance within 6–12 months, which is associated with mechanisms such as gene mutations, the lysosomal sequestration of TKIs, the activation of alternative pathways, and tumor heterogeneity [50,51,52].

Considering the real-world challenges in treating renal cell carcinoma, G6PD has emerged as a promising therapeutic target. Its inhibition, using nonspecific reagents such as dehydroepiandrosterone (DHEA) and 6-Aminonicotinamide (6-AN), has been reported in breast and cervical cancer cells and RCC, where it reduced the cell viability [53,54,55]. In our results, the 786-O cell line showed a greater sensitivity to the G6PDi-1 inhibitor in both viability and clonogenic assays compared to cabozantinib and lenvatinib, while, at lower concentrations, G6PDi-1 promoted cell viability in the ACHN. These opposite results may be related to the higher *G6PD* expression and activity in 786-O compared to ACHN [27] and suggest that ACHN has a lower dependency on G6PD-mediated metabolic pathways, which can impair the inhibitor effects on cells’ viability. Nonetheless, 786-O cells harbor a *VHL* mutation that stabilizes HIF and stimulates metabolic reprogramming, increasing the glucose-6-phosphate availability and NADPH production through the transcriptional regulation of *GAPDH* and *G6PD* [51].

Comparatively, the cell viability assay measures short-term cell survival based on metabolic activity and cytotoxic effects, whereas the clonogenic assay assesses long-term survival and the ability of a single cell to form a colony (at least 50 cells), which reflects cell cycle integrity, DNA repair capacity, and sustained proliferative potential [56]. We have used the resazurin assay, which is based on the reduction of resazurin (oxidized form) to resorufin (reduced fluorescent form) by metabolically active cells, allowing the estimation of cell viability. However, increases in resazurin reduction do not necessarily reflect increased cell proliferation and may instead indicate metabolic or redox adaptation under drug exposure [57]. In the migration assay, G6PDi-1 had a more potent effect than lenvatinib in both cell lines, and cabozantinib was the drug that most impaired the wound closure. Since the migratory cell capacity depends on cytoskeletal dynamics, focal adhesion, and matrix remodeling, it provides a rapid indicator of cell integrity [58].

While VEGF-targeted inhibitors were tested at identical concentrations to a enable direct comparison, G6PDi-1 was evaluated at different doses due to the lack of standardized dosing, which may represent a limitation of this study. Since G6PDi-1 is an experimental and selective inhibitor, it may exhibit distinct dose–response profiles, highlighting the need to define optimal dosing for therapeutic purposes. To date, G6PDi-1 has not been evaluated in RCC. However, studies in other mammalian cell types (HepG2, HCT116, T-cell leukemia cell lines, macrophages and neutrophils) demonstrated that it exerts significant biological effects by decreasing 6-phosphogluconate (6-pg) production, blocking electron transfer to NADP^+^, increasing the NADP^+^/NADPH ratio, inducing oxidative stress, and disrupting cytokine production [59]. In primary mouse astrocyte culture, G6PDi-1 directly affected the redox balance without causing significant toxicity [60], whereas, in platelets, it incurred mitochondrial damage, inducing platelet aggregation failure [61].

## 4. Materials and Methods

### 4.1. In Silico Analysis

*G6PD* gene expression was analyzed with the KM-Plotter platform (https://kmplot.com/analysis/. accessed on 8 July 2025), using two datasets (Gene-chip and Pan-cancer RNA-seq) [62]. The Gene-chip (277 normal, 556 tumors, and 58 metastasis) was evaluated through TNMplot (https://tnmplot.com/analysis/. accessed on 8 July 2025) and Targetgram. Differences between RCC subtypes and normal tissues were assessed with the Multi-gene analysis tool using the Pan-cancer RNA-seq dataset. The same dataset (Pan-cancer RNA-seq) was also used to evaluate the overall survival (OS) for ccRCC (*n* = 530) and pRCC (*n* = 288), with the median expression as the cut-off.

The relationship between *G6PD* expression and therapy response to lenvatinib and cabozantinib in the kidney cancer cell line was evaluated with the Receiver Operating Characteristic (ROC) plotter (https://rocplot.com. accessed on 19 December 2025), using the ‘Kidney cancer’ cell lines Depmap Dataset. The tool was set as follows: Response based on lower and upper tertiles of IC50, tissue ‘Kidney cancer’ and Depmap Dataset. The Mann–Whitney test and fold change (FC) inferred differences between responder and non-responder cells, while a larger area under the ROC curve (AUC) suggested better therapy response.

The DepMap tools (https://depmap.org/portal/. accessed on 19 December 2025) were used for dependency analysis and drug sensitivity dose curves. The first analysis used the datasets from CRISPR (DepMap Public 25Q3 + Score, Chronos) and RNAi (Achiles + DRIVE + MArcotte, DEMETER2) on ccRCC, non-cancerous (NC) and pRCC cell lines. The Chronos dependency score is based on data from a cell depletion assay. A lower Chronos score indicates a higher likelihood that the gene of interest is essential in a given cell line. A score of 0 (zero) indicates that a gene is not essential, whereas –1 is comparable to the median of all pan-essential genes. The drug sensitivity dose curves were analyzed with CTD^^2^ and PRISM Repurposing Secondary Screen datasets. Additional information regarding the cell line characteristics, the datasets used, and the analysis settings conducted in the DepMap Portal are provided in the Supplementary Materials S2.

### 4.2. Subjects and Immunohistochemistry (IHC)

A total of 121 RCC cases and 12 adjacent non-neoplastic kidney tissues were collected (2000–2021) from the Clinics Hospital, UFTM. Most of the cases were male (67.2%), with a median age of 62 years (mean of 60.8 ± 10.9 years, median = 62, range: 29 to 91 years old); 38.9% were overweight, 68.3% of them had co-morbidities such as diabetes mellitus, chronic kidney disease or hypertension and 52.7% were smokers and/or alcoholics. Most of the tumors (77.9%) were clear cell histological subtype (ccRCC); 17.6% were papillary (pRCC) and 4.6% were chromophobe (chRCC).

Tissue microarrays (TMAs) were constructed in duplicate. IHC was performed using the EasyLink One HRP Polymer Kit (EasyPath Diagnósticos, Indaiatuba, Brazil), with antigen retrieval in citrate buffer (pH 6.0) and overnight incubation at 4 °C with anti-G6PD antibody (1:500, sc-373886, Santa Cruz Biotechnology, Inc., Dallas, TX, USA). Sections were counterstained with hematoxylin. Two independent observers evaluated staining intensity (0–3) and percentage of positive cells (0–4). The final expression score was calculated by multiplying both parameters.

### 4.3. Cell Lines and Reagents

Functional assays were performed using commercial human cell lines, donated by Dra. Marilia De Freitas Calmon (Unesp, São José do Rio Preto-SP). 786-O, derived from a primary clear cell tumor (ATCC: CRL-1932), and ACHN (ATCC: CRL-1611) were cultured with RPMI 1640 and Eagle’s Minimum Essential Medium (EMEM) supplemented with 10% fetal bovine serum and 1% penicillin, respectively. Treatments were performed with the G6PD inhibitor G6PDi-1 (SML2980 Sigma-Aldrich Brasil LTDA, Cajamar, Brazil) and two anti-VEGFR tyrosine kinase inhibitors: cabozantinib (SML3914 Sigma-Aldrich Brasil LTDA, Cajamar, Brazil) and lenvatinib (SML3017 Sigma-Aldrich Brasil LTDA, Cajamar, Brazil). All data represent at least three independent experiments.

### 4.4. Cell Viability Assay (Resazurin)

The cells were seeded in quadruplicate in 96-well plates at a density of 6 × 10^3^ (786-O) and 7 × 10^3^ (ACHN) per well. After 24 h, both lines reached approximately 70% confluence and were treated with increasing concentrations of lenvatinib or cabozantinib (0, 1, 2, 4, 8, and 16 µM), or G6PDi-1 (0, 25, 50, 100, and 200 µM). After 24 and 48 h, 10 µL of resazurin/well (stock solution [440 µM]) was added. The cells were then incubated under the same culture conditions for 4 h, and the absorbance was measured (570 nm with a reference wavelength of 600 nm) using a spectrophotometer (INNO, LTEK Co., Seongnam-si, Republic of Korea). Culture medium supplemented with 3% dimethyl sulfoxide (DMSO) was used as the control group, with viability set at 100% in the comparative analyses.

### 4.5. Wound Healing Assay

Cells were seeded in 6-well plates (786-O: 2.5 × 10^5^; ACHN: 1 × 10^6^) and maintained in culture until ≥90% confluence was achieved (≅2 days). Then, the cell layer was scratched with a 200 µL pipette tip and treated with 8 µM lenvatinib or cabozantinib, or 105 µM of G6PDi-1 in basal medium without 10% fetal bovine serum. Doses were defined based on IC_50_ values obtained from the cell viability assay. At least four images of the wound area in each well were captured (0 and 24 h after treatment) and were analyzed using ImageJ version 1.54k, with the Wound healing size tool updated [63] to measure the area of the gap (in pixels). Culture medium supplemented with 1.5% DMSO was used as a control. The means and standard deviations of the gap areas, as well as the percentage of gap closure after 24 h, were calculated.

### 4.6. Clonogenic Assay

Cells were seeded in duplicate in 6-well plates (500 cells per well). After 24 h, treatments were performed with 4 µM lenvatinib or cabozantinib, or 52 µM G6PDi-1. Doses were defined based on IC_30_ values obtained from the cell viability assay. The cells were maintained for 10 days under culture conditions or until the control (complete medium supplemented with 1% DMSO) presented colonies with approximately 50 cells. The plates were fixed with ethanol 100% and stained with 0.5% crystal violet. The area occupied by the colonies was analyzed using the ImageJ plugging Colony Area [64].

### 4.7. Statistical Analysis

Data were analyzed using SPSS version 20 and GraphPad Prism version 9.0. Results were expressed as mean, median, standard deviation (SD), odds ratio (OR), fold change (FC), hazard ratio (HR), and 95% confidence interval (CI). Protein expression associations were tested using Chi-square or Fisher’s exact tests, and Mann–Whitney or Kruskal–Wallis tests. Survival curves were assessed using Kaplan–Meier curves and the log-rank test. *In vitro* data were analyzed through one-way ANOVA with Tukey’s post hoc test for multiple groups. The IC_30_ and IC_50_ values for the viability assay were calculated using the GraphPad Prism software version 9.0. Significance was set at *p* < 0.05.

## 5. Conclusions

In summary, our findings support a potential role for G6PD as a metabolic vulnerability associated with RCC aggressiveness and therapeutic response. By integrating patient samples, *in silico* analyses, and *in vitro* functional assays, we demonstrate that ccRCC cells are more dependent on the pentose phosphate pathway, turning them vulnerable to G6PD inhibition. High *G6PD* expression was associated with reduced therapeutic response to lenvatinib and, to a lesser extent, to cabozantinib across renal cancer cell lines. Notably, the inhibitor G6PDi-1 showed greater efficacy than lenvatinib and stronger antitumor effects than cabozantinib in 786-O cells. Nonetheless, sensitivity to VEGF-targeted drugs was cell line-dependent. These findings highlight the importance of metabolic profiling in therapeutic decision-making and suggest that targeting G6PD may represent a promising complementary strategy in RCC.

Nevertheless, this study has some limitations, such as the use of *in vitro* models, the use of a cohort local for the evaluation of protein expression, and the lack of direct metabolic pathway analyses. These limitations highlight the need for further validation in larger RCC cohorts, including pRCC and chRCC subtypes. Future mechanistic studies are required to better define the biological effects of G6PD inhibition, including oxidative stress-related pathways and downstream signaling associated with tumor cell proliferation, migration, and apoptosis.

## Data Availability

The original contributions presented in this study are included in the article/Supplementary Materials. Further inquiries can be directed to the corresponding author.

## Supplementary Materials

The following supporting information can be downloaded at: https://www.mdpi.com/article/10.3390/ijms27062844/s1.

## Abbreviations

The following abbreviations are used in this manuscript:

RCC	Renal cell carcinoma
ccRCC	Clear cell renal cell carcinoma
pRCC	Papillary renal cell carcinoma
chRCC	Chromophobe renal cell carcinoma
VEGF	Vascular endothelial growth factor
TKI	Tyrosine kinase inhibitor
G6PD	Glucose-6-phosphate dehydrogenase
IHC	Immunohistochemistry
