# Supplementary material for: Targeting G6PD (Glucose-6-Phosphate Dehydrogenase) as a Biomarker of Therapeutic Vulnerability in Renal Cell Carcinoma
Source: Int J Mol Sci. 2026 Mar 20;27(6):2844. doi: 10.3390/ijms27062844 (PMC13027040; doi:10.3390/ijms27062844)
Supplement: Supplementary file 1 [file ijms-27-02844-s001.zip › Supplementary Table S1.pdf]

Table 1 Percentage of positive cells for the G6PD immunolabeling according to the clinicopathological characteristics

Characteristics	Percentage of labeled cells					
	G6PD					
		≤50%	>50%			
	N (%)	n (%)	n (%)	p-value*	Odds ratio	CI 95%
**Age**						
<50 years	19 (15.7)	17 (89.5)	2 (10.5)	1.00	0.9	0.19 - 4.6
≥50 years	102(84.3)	92 (90.2)	10 (9.8)			
**Gender**						
Male	81 (66.9)	78 (96.3)	3 (3.7)	***0.002***	7.55	1.92 - 29.74
Female	40 (33.1)	31 (77.5)	9 (22.5)			
**Histopathology**						
ccCCR	93 (76.9)	89 (95.7)	4 (4.3)			
pCCR	22 (18.2)	16 (72.7)	6 (27.3)	***0.001***		
chCCR	6 (5.0)	4 (66.7)	2 (33.3)			
**Fuhrman grade**						
1	16 (13.2)	16 (100.0)	0 (0.0)			
2	57 (47.1)	54 (94.7)	3 (5.3)	0.058		
3	39 (32.2)	32 (82.1)	7 (17.9)			
4	9 (7.4)	7 (77.8)	2 (22.2)			
**Fuhrman grade**						
1 and 2	73 (60.3)	70 (95.9)	3 (4.1)	***0.012***	5.4	1.38 - 21.06
3 and 4	48 (39.7)	39 (81.2)	9 (18.8)			
**Tumor size**						
≤7 cm	64 (53.3)	62 (96.9)	2 (3.1)	***0.012***	6.74	1.41 - 32.24
>7 cm	56 (46.7)	46 (82.1)	10 (17.9)			
**Stage**						
I and II	81 (66.9)	75 (92.6)	6 (7.4)	0.19	2.2	0.66 - 7.33
III and IV	40 (33.1)	34 (85.0)	6 (15.0)			
**Necrosis**						
Absent	52 (43.0)	50 (96.2)	2 (3.8)	0.067	4.24	0.89 - 20.25
Present	69 (57.0)	59 (85.5)	10 (14.5)			
**Metastasis**						
No	107(91.5)	97 (90.7)	10 (9.3)	1.0	1.08	0.12 - 9.40
Yes	10 (8.5)	9 (90.0)	1 (10.0)			

ccRCC: Clear cell Renal Cell Carcinoma, pRCC: Papillary Renal Rell Carcinoma, chRCC: Chromophobe Renal Rell Carcinoma, CI: Confidence Interval, N: Number of cases. (*) Chi-square test or Fisher's test.
