# Supplementary figures and images for: Targeting G6PD (Glucose-6-Phosphate Dehydrogenase) as a Biomarker of Therapeutic Vulnerability in Renal Cell Carcinoma
Source: Int J Mol Sci. 2026 Mar 20;27(6):2844. doi: 10.3390/ijms27062844 (PMC13027040; doi:10.3390/ijms27062844)

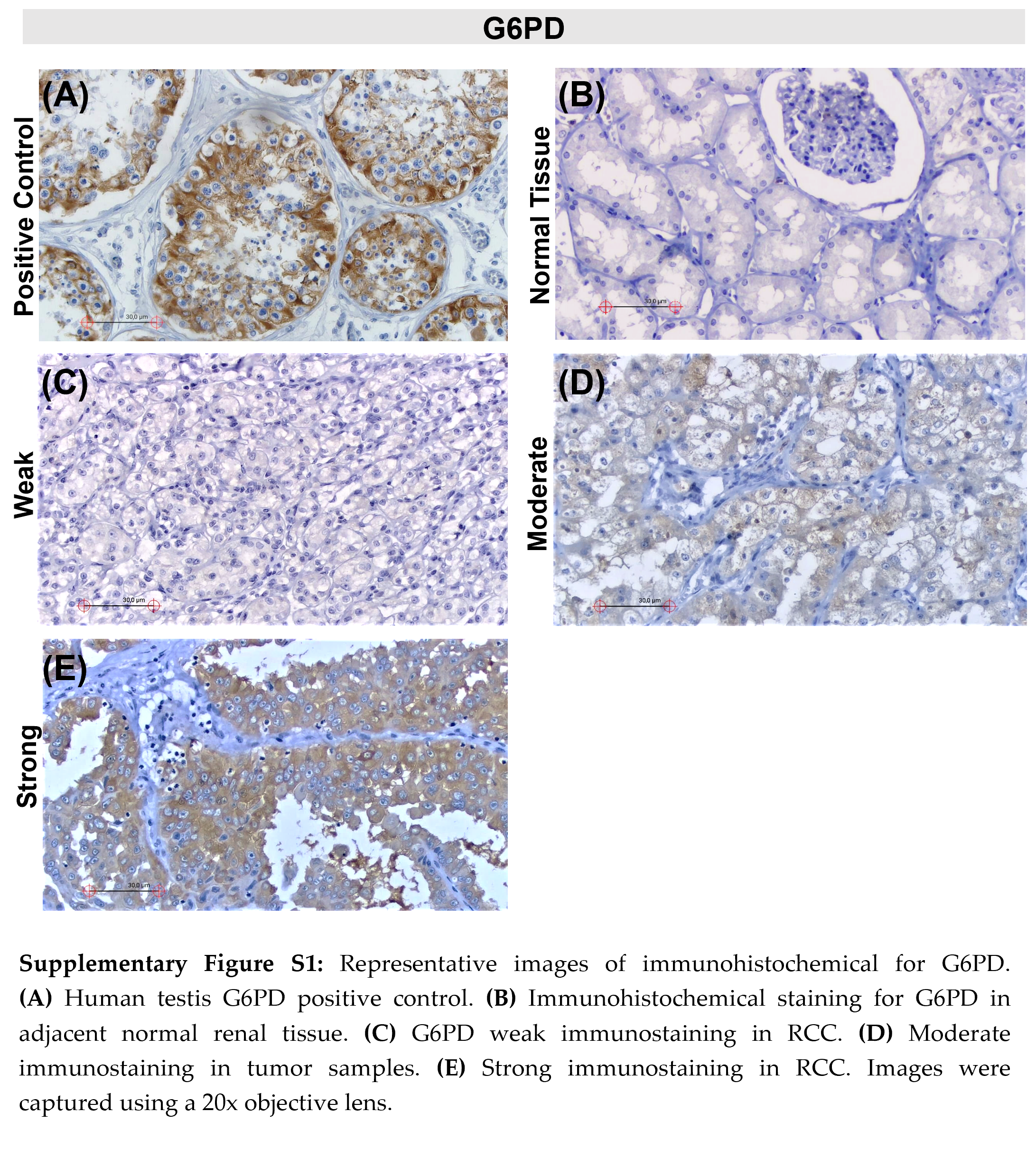

Supplement: Supplementary file 1 [file ijms-27-02844-s001.zip › Supplementary Figure S1.png]

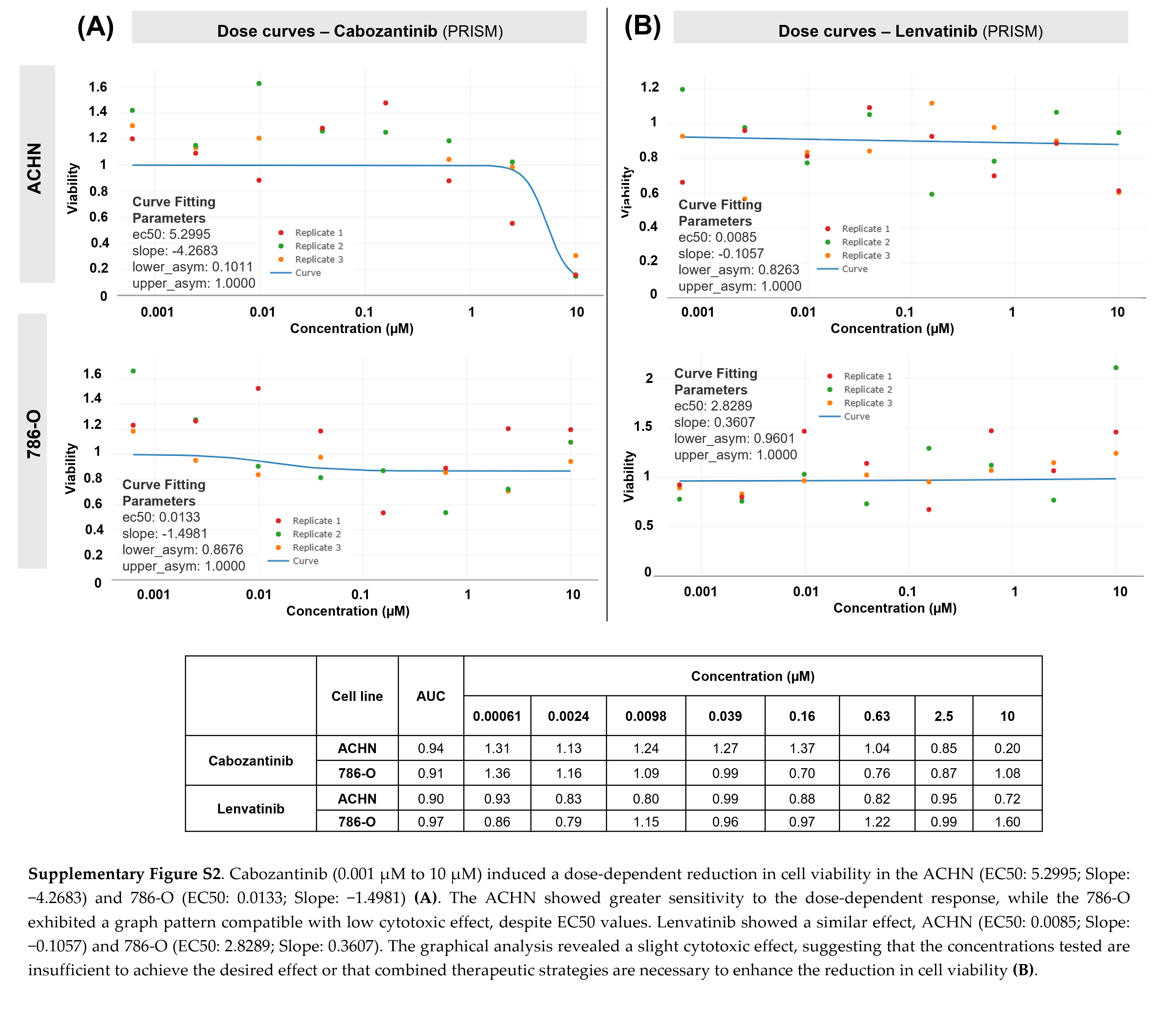

Supplement: Supplementary file 1 [file ijms-27-02844-s001.zip › Supplementary Figure S2.png]

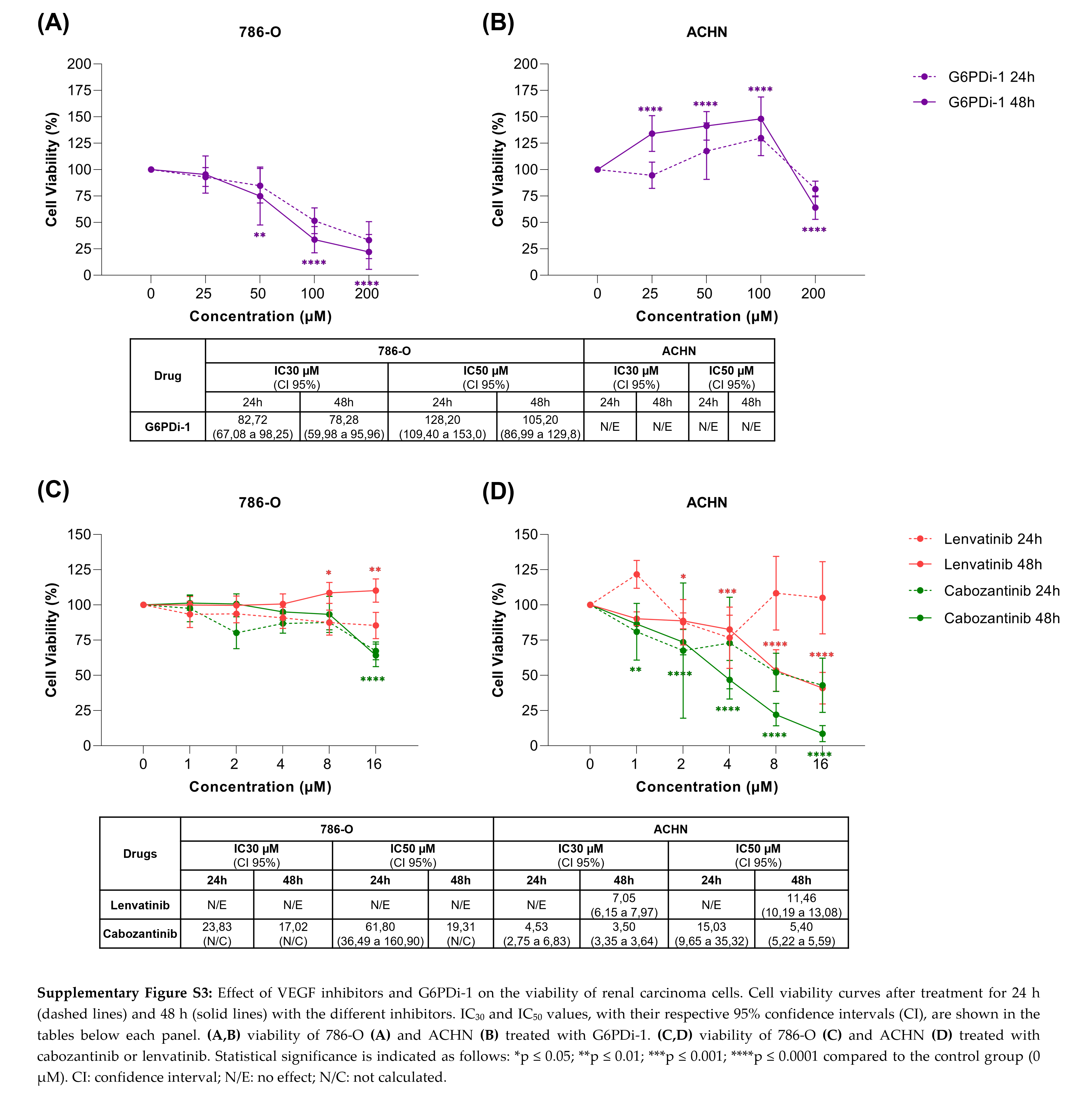

Supplement: Supplementary file 1 [file ijms-27-02844-s001.zip › Supplementary Figure S3.png]
